# Influence of skin-to-skin contact on breastfeeding: results of the Mexican National Survey of Demographic Dynamics, 2018

**DOI:** 10.1186/s13006-022-00489-2

**Published:** 2022-07-07

**Authors:** Clara Luz Sampieri, Karina Gutiérrez Fragoso, Daniel Córdoba-Suárez, Roberto Zenteno-Cuevas, Hilda Montero

**Affiliations:** 1 Instituto de Salud Pública, Universidad Veracruzana, Av. Luis Castelazo Ayala S/N, Col. Industrial Ánimas, 91190 Xalapa, Veracruz México; 2 Instituto Tecnológico Superior del Oriente del Estado de Hidalgo, ITESA. Carretera Apan-Tepeapulco km 3.5, Las Peñitas, 43900 Apan, Hidalgo México

**Keywords:** Breastfeeding, Skin-to-skin contact, Mother-baby pair, Mexico

## Abstract

**Background:**

Skin-to-skin contact and breastfeeding initiation within the first hour after birth are key recommendations to promote breastfeeding. In Mexico, the National Survey of Demographic Dynamics 2018, known by its Spanish acronym ENADID, collected information about breastfeeding practices. The ENADID survey is probabilistic and allows results to be generalized to the entire population in Mexico.

**Methods:**

Information from a public database featuring 26,587 mother-baby pairs was analyzed by proportions, means and associations, as well as machine learning methods, to conduct a comparison among the pairs according to immediate skin-to-skin contact after delivery status.

**Results:**

Skin-to-skin contact was described by 78.7% of the mothers and was associated with receiving an explanation regarding how to give breastmilk or the breast to the baby immediately following birth [Odds ratio (OR) 6.46; 95% Confidence Interval (CI) 6.02, 6.97], initiating breastfeeding in the first hour of life (OR 2.01; 95% CI (1.84, 2.18) and a breastfeeding duration of ≥ 6 months (OR 1.16; 95% CI 1.08, 1.25). The breastfeeding duration, in days, was greater in the group with skin-to-skin contact than in the group without skin contact.

**Conclusions:**

In Mexico, immediate and uninterrupted skin-to-skin contact between newborns and their mothers should be facilitated. Support should be provided to mothers to favor skin-to-skin contact and breasting initiation during the first hour of life, ideally through an empathic explanation by trained health personnel. Future research should focus on the evaluation of strategies to modify maternity services to facilitate immediate skin-to-skin contact after delivery and develop training programs for health personnel to support the initiation of breastfeeding during the first hour of life.

**Supplementary Information:**

The online version contains supplementary material available at 10.1186/s13006-022-00489-2.

## Background

Breastfeeding should be understood as a continuation of the intrauterine dialog through the placenta and umbilical cord, and breastfeeding can save lives and reduce mother-infant pair morbidity [1]. The World Health Organization (WHO) and the United Nations Children’s Fund (UNICEF) recommend the initiation of breastfeeding within the first hour of life and exclusive breastfeeding until six months of age [2]. Thereafter, the addition of safe, nutritious, and age-appropriate complementary foods with continued breastfeeding is recommended up to two years of age and beyond. In 2015, the WHO and UNICEF began the re-evaluation of the Baby-Friendly Hospital Initiative (BFHI) [2]. The 2018 version of the Ten Steps of the BFHI includes two critical management procedures and eight key clinical practices. Step 4 of the BFHI refers to “facilitating immediate and uninterrupted skin-to-skin contact and supporting mothers in initiating breastfeeding as soon as possible after birth” as a key clinical practice to successful breastfeeding [2].

In Mexico, the National Survey of Demographic Dynamics 2014 (ENADID, by its Spanish acronym) estimated that 11% of infants were exclusively breastfed and that the mean duration of breastfeeding was 8.8 months [3]; four years later, the ENADID 2018 reported increases to 12.9% of infants and 9.8 months, respectively [4]. The ENADID 2018, unlike the previous survey, included a question about skin-to-skin contact immediately after birth [5].

In Mexico, the Official Mexican Norm NOM-007-SSA2-2016, regarding attention to mothers during pregnancy, birth and puerperium and to newborns, indicates that the initiation of breastfeeding should occur within the first 30 min of a newborn’s life, for mothers and newborns whose health conditions allow it [6]. The objective of this study was to determine the association of skin-to-skin contact between mothers and their newborns immediately following birth, with the initiation of breastfeeding during the first hour of life, a breastfeeding duration of ≥ 6 months, and the introduction of breastmilk substitutes. To pursue this objective, data from the ENADID 2018 were used.

## Methods

### Study design

This was a descriptive study in which the data produced by the ENADID 2018 were analyzed [5]. The ENADID survey was designed, validated, applied and analyzed by the Mexican National Institute of Statistics and Geography (INEGI, by its Spanish acronym); public data from this survey are available for the years 1992, 1997, 2006, 2009, 2014 and 2018 [5]. The ENADID survey aims to provide statistical information about demographic dynamics, fertility, mortality, and migration, as well as other issues related to the population, households, and housing; this survey contains two questionnaires: one for the household and another for the mother [5]. The validation process of the information collected by the ENADID is carried out by three procedures: field validation, primary validation and automatic validation [5]. Furthermore, a statistical test for validation of the ENADID 2018 was applied in a sample, obtaining complete information for questionnaires from 2684 households and 2859 mother-baby pairs [5].

### Participants

All women between 15 and 54 years of age with habitual residence who reported having had their last pregnancy between January 2013 and the date of the ENADID 2018 interview and who had given birth to a live-born child were included in this study (*N* = 26,587 mother-baby pairs) [5].

### Setting and data collection

The ENADID 2018 survey collected data by direct interviews in the participants’ homes in Mexico from the 13th of August to the 5th of October 2018 and had national coverage with its sample size of 119,800 households [5].

### Data analysis

The groups from the ENADID 2018 survey included for analysis were mother-baby pairs classified into two groups: those with skin-to-skin contact immediately after birth and those without skin-to-skin contact immediately after birth. These groups were defined by the following question: “After birth, did they place the baby on your breast in direct contact with your skin?” Direct skin-to-skin contact was defined by the ENADID 2018 “as the practice of placing the newborn skin-to-skin on the breast of the mother immediately after birth, with the aim of strengthening the bond between the two. It does not imply breastfeeding” [5]. Note that the ENADID 2018 survey did not specify, in minutes, the term “immediately after birth”.

The variables of the ENADID 2018 that were analyzed included the following: sociodemographic stratum, defined as the classification of a household according to certain socioeconomic characteristics of the inhabitants, as well as the physical characteristics and equipment of the household, expressed through indicators constructed with information of the population and the household census of Mexico as low/medium–low/medium–high/high; the size of the locality, reported as urban (≥ 2500 inhabitants) or rural (< 2500 inhabitants); the conditions of activity, defined as an economically active population or an economically inactive population; partner status, defined as married or in a civil union/not having a partner (single, widowed, separated or divorced); affiliation with health institutions, grouped as having an affiliation or not having an affiliation; and the number of prenatal visits [5].

Additionally, analyzed variables of the ENADID 2018 were delimited by the following questions: “After delivery, did they explain to you how to give breastmilk or the breast to the baby?” (received an explanation of breastfeeding after delivery). Note that the ENADID 2018 survey did not specify who provided this information, how long after birth this explanation occurred and what the explanation consisted of. “Did you give breastmilk or the breast to the baby?”; an answer of “Yes” was considered as ever breastfeeding, and an answer of “No” was considered as never breastfeeding. “Why did you not give breastmilk or the breast to the baby?” (reason for not breastfeeding); the answer options were never having milk/infant rejection/maternal illness/infant illness/a doctor recommended the use of infant formula/the baby died at the time of birth/other reason/unknown. “How long after birth did you begin to give breastmilk or the breast to the baby?” (initiation of breastfeeding). “For how long are you giving (or did you give) breastmilk or the breast to the baby?” (duration of breastfeeding), which was grouped into less than a day, 1–29 days, 1–5 months, 6–12 months, 13–24 months and more than 25 months. The duration of breastfeeding in days was calculated by multiplying the number of months by 30 or the number of years by 365; in participants with a breastfeeding duration of ≤ 23 h, a value of zero was assigned. “At how many days or months after the birth did you began to give water or tea/infant formula, powdered milk, bovine milk, juices, etc., broths/purees/*atole*, cereals, tortillas, or bread/eggs?” (introduction of breastmilk substitutes). Note that *atole* is a beverage of water or milk made with corn. The rest of the variables analyzed in this study are described in the Additional file 1 [5].

Categorical variables are summarized using absolute frequencies and percentages. Continuous variables are expressed as medians and the interquartile range for nonnormal distributions and as means and standard deviations (SD) for normal distributions. Proportions and means were compared using the chi-square test and Student’s t test, respectively. The proportions of exposure to breastmilk substitutes of surviving infants were compared and grouped into durations of less than a day, 2–4 days and 5 days-5 months and ≥ 6 months. Associations were determined by odds ratios. A *p* value of ≤ 0.05 was considered statistically significant. IBM-SPSS Statistics version 23 was used for analysis.

For the machine learning methods analysis, groups were delimited by the question “Did you give breastmilk or the breast to the baby?”, which eliminated the participants of infants who died at the time of birth (*n* = 45), at the time of the interview they were still breastfeeding (*n* = 5303), participants who in some question have some missing value (*n* = 938) and those who unknown breastfeeding duration (*n* = 76); two groups were obtained: those who had ever breastfed (*N* = 18,564) and those who had never breastfed (*N* = 1661). For the analysis of the evaluation of attributes, through the program Weka, version 3.8.4, selection algorithms that were generated by supervised learning were used. These were applied to assess the importance of the variables; information regarding these methods can be found in the publication by Al Janabi and Rusul [7]. For analysis of the methods of classification, the models of Bayesian networks and decision trees were considered using the algorithms implemented by Weka (Bayes Net and J48), reporting the results without the reduction of dimensionality. The structure of the Bayesian network was generated through data learning [8, 9]. Induction of the decision tree was conducted through the learning process from a set of training examples with labels of known classes, in which an assessed attribute at each depth level was selected based on the criterion of the proportion of information gain [10]. The detailed machine learning methodology employed is described in the Additional file 1.

## Results

### General characteristics of the participants

Information from a public database including 26,587 mother-baby pairs was analyzed, which is representative of the entire population in Mexico. A total of 20,916 (78.7%) mother-baby pairs reported having practiced skin-to-skin contact immediately after birth.

The mean (± SD) age of the mothers was 28.75 (6.725) and 28.54 (6.776) years for the groups with and without skin-to-skin contact, respectively. The general characteristics of the groups are presented in Table 1. In general, most of the participants were found in the low and medium–low sociodemographic strata, lived in an urban locality, did not consider themselves indigenous and had a partner. Most of the population met the minimum conditions for the management of breastfeeding, e.g., they did not have dirt flooring in the home, and they had access to piped water and a refrigerator (Table 1).

**Table 1:** General characteristics of the mother-baby pairs, data from the Mexican National Survey of Demographic Dynamics 2018, *N* = 26,587

Characteristic	Skin-to-skin contact immediately after birth	
	**Yes** ***N* = 20,916**	**No** ***N* = 5671**
	***n* (%)**	***n* (%)**
**Sociodemographic stratum**		
Low	5098 (24.4)	1195 (21.1)
Medium–low	11,397 (54.5)	3141 (55.4)
Medium–high	3206 (15.3)	976 (17.2)
High	1215 (5.8)	359 (6.3)
**Locality**		
Urban	14,793 (70.7)	4196 (74.0)
Rural	6123 (29.3)	1475 (26.0)
**Considering themselves indigenous**		
Yes	8163 (39.0)	2048 (36.1)
No	12,753 (61.0)	3623 (63.9)
**Having a partner**		
Yes	17,276 (82.6)	4611 (81.4)
No	3640 (17.4)	1060 (18.7)
**Education level**		
None	290 (1.4)	64 (1.1)
≤ Primary	2906 (13.9)	689 (12.2)
> Primary	17,720 (84.7)	4918 (86.7)
**Condition of economic activity**		
Active	9385 (44.9)	2603 (45.9)
Inactive	11,531 (55.1)	3068 (54.1)
**Affiliation to health institution**		
Yes	18,545 (88.7)	5001 (88.2)
No	2371 (11.3)	670 (11.8)
**House floor material**		
Dirt	778 (3.7)	207 (3.7)
Cement or firm	11,903 (56.9)	3078 (54.3)
Wood, mosaic or other	8235 (39.4)	2386 (42.1)
**Piped water to the house or plot of land**		
Yes	19,542 (93.4)	5324 (93.9)
No	1374 (6.6)	347 (6.1)
**Refrigerator in the house**		
Yes	17,966 (85.9)	4959 (87.4)
No	2950 (14.1)	712 (12.6)

*n*, Number of participants presenting the characteristic of interest. The percentage corresponds to the column

The characteristics of the prenatal, birth and postpartum periods of the groups with or without skin-to-skin contact are presented in Table 2. Overall, a very low percentage of participants reported the absence of prenatal visits, most of the deliveries took place in a medical unit, and 12,029 (45.4%) births were by cesarean section. A higher percentage of women who had skin-to-skin contact immediately after birth (90.8%) received an explanation about how to give their baby breastmilk or the breast compared to those who did not have skin-to-skin contact (60.3%). Ever breastfeeding was reported by 94.5% of the skin-to-skin-contact group, compared to 82.1% of the group who did not have skin-to-skin contact. In general, mothers selected the answer options “never had milk” and “infant rejection” as the reasons behind never breastfeeding (Table 2).

**Table 2:** Prenatal visits, general characteristics of the birth and postpartum periods, data from the Mexican National Survey of Demographic Dynamics 2018

Characteristic	Skin-to-skin contact immediately after birth	
	**Yes**	**No**
	***n* (%)**	***n* (%)**
**Number of prenatal visits**		
None	24 (0.1)	8 (0.1)
1–2	211 (1.0)	94 (1.7)
3–7	5588 (26.7)	1491 (26.3)
≥ 8	14,868 (71.1)	3746 (66.1)
Not specified	225 (1.1)	332 (5.9)
**Delivery in a medical unit**		
Yes	20,239 (96.8)	5349 (94.3)
No	655 (3.1)	186 (3.3)
Not specified	22 (0.1)	136 (2.4)
**Type of delivery**		
Vaginal birth	12,049 (57.6)	2106 (37.1)
Cesarean section	8867 (42.4)	3162 (55.7)
Not specified	0 (0)	403 (7.1)
**Receiving an explanation of breastfeeding after delivery**		
Yes	18,983 (90.8)	3419 (60.3)
No	1933 (9.2)	2252 (39.7)
**Ever breastfeed**		
Yes	19,772 (94.5)	4656 (82.1)
No	1140 (5.5)	582 (10.3)
Unknown	4 (0)	433 (7.6)
**Motives for never breastfeed**		
Never had milk	476 (41.8)	171 (29.4)
Infant rejection	276 (24.2)	97 (16.7)
Maternal illness	133 (11.7)	90 (15.5)
Infant illness	63 (5.5)	93 (16.0)
Doctor recommended the use of formula	49 (4.3)	27 (4.6)
Baby died at the time of birth	13 (1.1)	42 (7.2)
Another reason/unknown	130 (11.4)	62 (10.7)
**Initiation of breastfeeding**		
First hour	5259 (26.6)	726 (15.6)
Second hour-23 h	12,227 (61.8)	2414 (51.8)
Second day	1078 (5.5)	452 (9.7)
Third day	414 (2.1)	282 (6.1)
Fourth day	287 (1.5)	241 (5.2)
From the fifth day onwards	309 (1.6)	338 (7.3)
Unknown	198 (1.0)	203 (4.4)
**Duration of breastfeeding**		
Less than a day	127 (0.8)	31 (0.8)
1–29 days	901 (5.8)	242 (6.6)
1–5 months	4093 (26.5)	1063 (29.1)
6–12 months	7436 (48.0)	1611 (44.1)
13–24 months	2398 (15.5)	581 (15.9)
More than 25 months	456 (3.0)	110 (3.0)
Unknown	62 (0.4)	14 (0.4)

*n,* Number of participants presenting the characteristic of interest. The percentage corresponds to the column

### Exposure to breastmilk substitutes

The percentages of exposure to breastmilk substitutes by groups with and without skin-to-skin contact, with data from the ENADID 2018, are presented in Table 3. The results indicated that all breastmilk substitutes included in the ENADID 2018 questionnaire were offered to the newborn from the day of birth in both groups. In general, a total of 15,329 (57.8%) and 18,190 (68.6%) surviving infants ≤ 5 months old were exposed to infant formula, powdered milk or bovine milk, and water, respectively. A higher proportion of surviving infants aged ≤ 5 months were exposed to formula, powdered milk or bovine milk in the group without skin-to-skin contact than in the group with skin-to-skin contact (*P* = 0.0001). On the other hand, a higher proportion of surviving infants aged ≤ 5 months were exposed to water or tea in the group with skin-to-skin contact than in the group without skin-to-skin contact (*P* = 0.0001). Similar proportions of surviving infants aged ≤ 5 months between the compared groups were exposed to juices, broths, purees, *atole*, cereals, tortillas, bread, and eggs (*P* > 0.05).

**Table 3:** Age of surviving infants at the time of introduction to breastmilk substitutes, data from the Mexican National Survey of Demographic Dynamics 2018, *N* = 26,532

Breastmilk substitute/ age of introduction	Skin-to-skin contact immediately after birth	
	**Yes** ***N* = 20,903**	**No** ***N* = 5629**
	***n* (%)**	***n* (%)**
**Formula, powdered milk or bovine milk**		
Less than a day	1901 (9.1)	634 (11.3)
2–4 days	2729 (13.1)	902 (16.0)
5 days-5 months	7379 (35.3)	1784 (31.7)
≥ 6 months	5562 (26.6)	1127 (20.0)
Has not been offered	556 (2.7)	164 (2.9)
Unknown	2776 (13.3)	1018 (18.1)
**Water or tea**		
Less than a day	442 (2.1)	113 (2.0)
2–4 days	1230 (5.9)	262 (4.7)
5 days-5 months	13,071(62.5)	3072 (54.6)
≥ 6 months	5251 (25.1)	1443 (25.6)
Has not been offered	886 (4.2)	291 (5.2)
Unknown	23 (0.1)	448 (8.0)
**Juices or broths**		
Less than a day	277 (1.3)	76 (1.4)
2–4 days	1230 (5.9)	262 (4.7)
5 days-5 months	6527 (31.2)	1546 (27.5)
≥ 6 months	12,353 (59.1)	3013 (53.5)
Has not been offered	833 (4.0)	286 (5.1)
Unknown	879 (4.2)	696 (12.4)
**Purees**		
Less than a day	336 (1.6)	77 (1.4)
2–4 days	18 (0.1)	9 (0.2)
5 days-5 months	5859 (28.0)	1440 (25.6)
≥ 6 months	12,473 (59.7)	3027 (53.8)
Has not been offered	831 (4.0)	282 (5.0)
Unknown	1386 (6.6)	794 (14.1)
**Atole, cereals, tortillas, or bread**		
Less than a day	343 (1.6)	79 (1.4)
2–4 days	13 (0.1)	11 (0.2)
5 days-5 months	2596 (12.4)	599 (10.6)
≥ 6 months	15,620 (74.7)	3810 (67.7)
Has not been offered	841 (4.0)	291 (5.2)
Unknown	1490 (7.1)	839 (14.9)
**Egg**		
Less than a day	414 (2.0)	96 (1.7)
2–4 days	19 (0.1)	2 (0.0)
5 days-5 months	1162 (5.6)	278 (4.9)
≥ 6 months	15,852 (75.8)	3856 (68.5)
Has not been offered	856 (4.1)	296 (5.3)
Unknown	2600 (12.4)	1101 (19.6)

*n*, Number of participants presenting the characteristic of interest. The percentage corresponds to the column

### Association of the characteristics of participants and hospital practices with breastfeeding

Skin-to-skin contact was associated with vaginal birth (OR 2.04; 95% CI 95 1.91, 2.16); receiving an explanation of breastfeeding after delivery (OR 6.46; 95% CI 6.02, 6.97); the initiation of breastfeeding in the first hour of life (OR 2.01; 95% CI 1.84, 2.18); ever breastfeeding (OR 2.16; 95% CI 1.94, 2.40); and a breastfeeding duration of ≥ 6 months (OR 1.16; 95% CI 1.08, 1.25). The breastfeeding duration in days was greater in the group with skin-to-skin contact [median 240 (P25 90, P75 365)] than in the group without skin contact [median 210 (P25 90, P75 365)] (*P* value < 0.05). Women who considered themselves indigenous had a longer breastfeeding duration in days [median 270 (P25 120, P75 365)] compared to those who did not consider themselves indigenous [median 180 (P25 90, P75 365)] (*P* value < 0.05). On the other hand, the duration of breastfeeding in days for participants in the low sociodemographic stratum [median 365 (P25 180, P75 480)] was greater than those of participants in the medium–low stratum [median 210 (P25 90, P75 365)] (*P* value < 0.05), medium–high stratum [median 180 (P25 90, P75 360)] (*P* value < 0.05) and high stratum [median 180 (P25 90, P75 300)] (*P* value < 0.05).

The analysis through Bayesian networks and decision trees of the participants that ever breastfed or never breastfed, according to skin-to-skin contact status, indicated that there was a relationship of probabilistic dependence between skin-to-skin contact and receiving an explanation of breastfeeding after delivery. Receiving an explanation of how to give breast milk or breast to the baby after birth is an important attribute regarding skin-to-skin status in both groups (Additional files 2, 3, 4, 5 and 6).

## Discussion

To our knowledge, using data from the ENADID 2018 survey, this is the first study that demonstrated an association between skin-to-skin contact in the mother-baby pair immediately after birth with breastfeeding initiation in the first hour of life, a breastfeeding duration of ≥ 6 months and receiving an explanation about breastfeeding immediately after birth. These findings are supported by machine learning methods, offering visual models to facilitate the support and understanding of the phenomenon. Bayesian network analysis showed that in mother-baby pairs who had ever breastfed, there was a probabilistic dependence between skin-to-skin contact with the initiation of breastfeeding, the duration of breastfeeding and receiving an explanation about breastfeeding immediately after birth. Interestingly, the machine learning methods also showed a probabilistic dependence between the type of delivery with breastfeeding initiation and breastfeeding duration. Furthermore, infant formula, powdered milk or bovine milk supplementation was higher in the group without skin-to-skin contact than in the group with skin-to-skin contact. The results are difficult to compare because the previous survey, the ENADID 2014, did not include a question about skin-to-skin contact between newborns and mothers.

There is evidence in Mexico that the information about breastfeeding provided by health personnel to mothers is not enough to support them in initiating or continuing with any type of breastfeeding. Hernández-Cordero and colleagues reported that in a sample of 543 women, 34.7% supplemented with infant formula at the hospital [11]. A rate of 44.8% of exclusive breastfeeding at 1 month postpartum was estimated, as well as a higher timely initiation of breastfeeding in mothers with vaginal delivery and those who received information during pregnancy [11]. Although exclusive breastfeeding at 1 month postpartum was associated with mothers who received more information about breastfeeding during pregnancy, the perception of having an insufficient supply of breastmilk and the belief that infant formula is recommended persisted [11]. Health personnel know about the benefits of breastfeeding but have not been adequately trained to solve practical problems associated with breastfeeding [11]. In this context, a pioneering study carried out in a maternity service in Mexico showed that breastfeeding guidance during the hospital stay, including practical breastfeeding advice from a trained nurse, was associated with higher full breastfeeding rates in the group with breastfeeding guidance, compared to the group who had not received guidance [12].

Research has shown that the implementation of evidence-based guidelines that improve hospital practices favors breastfeeding rates. In a hospital on the Texas–Mexico border, a plan–do–study–act model carried out over a 2-year period successfully implemented the practice of skin-to-skin contact between newborns and mothers in the operating room, maintaining a 25% cesarean birth rate, substantial improvements in breastfeeding initiation and exclusive breastfeeding rates at hospital discharge [13].

In Mexico, it has been reported that study programs for health personnel do not provide training in counseling mothers to support breastfeeding [14]. Health personnel in Mexico should be supported to increase breastfeeding knowledge, develop practical skills and change attitudes [14]. Theoretical and practical training in breastfeeding should be a fundamental and indispensable component in all university degrees in health sciences [14]. Although continuous education programs in breastfeeding have been proposed in Mexico, their impact is unknown, and the indicators are still below the minimum recommended by the WHO [15]. Moreover, it has been reported that almost 60% of health personnel lack knowledge regarding the International Code of Marketing of Breastmilk Substitutes, subscribed by Mexico in 1981 as member states of the WHO, and that violations are common [16].

In Mexico, it is also necessary to promote breastfeeding in mass media and popular events, such as World Breastfeeding Week, including the participation of civil society and professional organizations free from commercial conflicts of interest [17, 18]. Operational research, monitoring, and evaluation are also essential to promote breastfeeding rates in Mexico [17].

Breastfeeding counseling is an important approach to improving global breastfeeding practices [19]. The WHO and UNICEF guidelines, “Implementation guidance on counseling women to improve breastfeeding practices”, outline six key recommendations to ensure that breastfeeding counseling is provided as follows: 1) to all pregnant women and mothers with young children; 2) in both the antenatal period and the postnatal period and up to 24 months or longer; 3) at least six times, and additionally as needed; 4) through face-to-face counseling, or additionally, through telephone or other remote modes of counseling in certain contexts; 5) as a continuum of care, by appropriately trained health care professionals and community-based lay and peer breastfeeding counsellors; and 6) as anticipatory guidance to address important challenges and contexts for breastfeeding, in addition to promoting skills, competencies, and confidence among mothers [19].

In addition, the UNICEF UK Baby Friendly Initiative states that the conversations in the immediate postpartum period between health personnel and mothers who breastfeed should be focused on closeness, and the meaning of responsive feeding and how to make it work for them can be part of ongoing discussions [20]. In addition, it should be explained to mothers that they cannot overfeed their baby and that maternal breastmilk can promote comfort and rest for the baby, as well as “food” [20]. As a safety issue, upon release from the hospital, parents should understand the signals regarding whether their baby is getting enough breastmilk [20]. Although antenatal breastfeeding education contributed to the substantial increase in the initiation of breastfeeding, at hospital discharge, new parents should be provided with resources that favor breastfeeding establishment, including support from breastfeeding consultants, inclusion in a support group and early follow-up with a health provider that is competent in breastfeeding [21].

Evidence has shown that in normal neonates, breastfeeding reflexes are strong at birth; indeed, preterm babies are capable of breastfeeding efficiently [22, 23]. Consequently, hospital practices, such as the separation of the mother-baby pair, can affect the establishment of breastfeeding [2]. In Mexico, the social determinants of health require greater articulation and concerted actions in different sectors, such as education, labor, and the development of social and indigenous people [24]. These articulations and actions should consider care of the mother-baby pair as a continuum, from the prenatal stage to natural weaning, with strict adherence to the International Code of Marketing of Breastmilk Substitutes.

Infant formula supplementation before hospital discharge is associated with a reduction in breastfeeding duration, undermining maternal confidence, affecting the natural microbiome and increasing the risk of early weaning [25, 26]. In addition to supplementation with infant formula, this study demonstrated that supplementation during the first day of life occurs with other breastmilk substitutes, such as water, tea, egg, *atole*, cereals, tortillas, bread, purees, juices and broths. Previous studies in Mexico have shown the introduction of breastmilk substitutes before the WHO recommendations, with a great variety of foods, such as coffee, pork, fish, and soft drinks [27, 28].

Hospital barriers to breastfeeding must be addressed to favor the maximum state of health of the mother-baby pair, and this is of particular importance during the COVID-19 pandemic. According to Lubbe and collaborators, “current evidence states that the coronavirus is not transmitted via breastmilk”, and therefore, to support breastfeeding during the current pandemic, it is crucial to understand the clinical characteristics of COVID-19 and the protective properties of breastfeeding, including practicing skin-to-skin contact between newborns and mothers [29]. In the context of the COVID-19 pandemic, the separation of mothers with suspected or confirmed SARS-CoV-2 from their infants after birth can cause an excess of illnesses and preventable deaths [30], as well as costs that impact the health system and families alike.

### Strengths and limitations

To our knowledge, this is the first report in Mexico that can be generalized at the national level about the association of skin-to-skin contact between mothers and the babies immediately after birth with breastfeeding initiation in the first hour of life; a breastfeeding duration of ≥ 6 months; and receiving an explanation about breastfeeding. The strength of this study is the sample design, which was probabilistic and included a considerable number of participants (*N* = 26,587). The results represent support for public health interventions in Mexico to promote breastfeeding.

The lack of a definition in time (minutes) of the concept "immediately after delivery" relating to the time elapsed between birth and the start of skin-to-skin contact between newborns and their mothers, as well the lack of data if this was uninterrupted, are a considerable limitation of this study, apart from possible memory bias. Other limitations of this study are the lack of specification about timing, personal responsibility for providing an explanation about how to give the baby breastmilk or the breast after delivery, explanation content and employed resources. We considered that the lack of specifications due to the instruments used to collect the information had an implication on the results.

## Conclusions

In Mexico, the practice of skin-to-skin contact between babies and their mothers, as well as providing an explanation about breastfeeding immediately after birth, are key to supporting breastfeeding. Conversations with mothers in the immediate postpartum period should ideally be adapted and carried out by trained personnel to favor the early initiation of breastfeeding. Uninterrupted skin-to-skin contact for mother-baby pairs should ideally be initiated as soon as possible after delivery. Both practices are feasible interventions that can be implemented by adapting maternity services, training health personnel and empowering mothers, among other processes. In Mexico, research is needed on the evaluation of strategies to adapt maternity services to facilitate uninterrupted immediate skin-to-skin contact after delivery between newborns and their mothers, as well developing breastfeeding training programs for health personnel to support mothers in initiating breastfeeding during the first hour of life.

## Supplementary Information

**Additional file 1. **Additional definitions of ENADID 2018 variables analyzed and detailed machine learning methodology.

**Additional file 2. **Ever breastfed group Bayesian network, data from the Mexican National Survey of Demographic Dynamics 2018. The analysis through Bayesian networks in mother-baby pairs that ever breastfed, showed a relationship of probabilistic dependence between the skin-to-skin contact and receiving an explanation of breastfeeding after delivery, initiation of breastfeeding and breastfeeding. There is a probabilistic relationship of skin-to-skin contact with delivery type, maternal age, sociodemographic stratum, considering themselves indigenous, locality, and education level. Two attributes converge probabilistically for the initiation of breastfeeding: delivery type and skin-to-skin contact, while four attributes converge for the duration of breastfeeding: sociodemographic stratum, indigenous self-adscription, delivery type and skin-to-skin contact.

**Additional file 3. **Ever breastfed group decision tree, data from the Mexican National Survey of Demographic Dynamics 2018. Analysis of the decision trees of mother-baby pairs that ever breastfed, had a depth of four levels from the root node, skin-to-skin contact, and 11 nodes, including six terminal nodes. The variable that produces the first split in the decision tree is receiving an explanation of breastfeeding after delivery; initiation of breastfeeding appears at the second division, followed by delivery type and the duration of breastfeeding.

**Additional file 4. **Never breastfed group Bayesian network, data from the Mexican National Survey of Demographic Dynamics 2018. The analysis through Bayesian networks in mother-baby pairs that never breastfed, skin-to-skin contact and receiving an explanation of breastfeeding after delivery were directly related to the motive for no breastfed.

**Additional file 5. **Never breastfed group decision tree, data from the Mexican National Survey of Demographic Dynamics 2018. The analysis of the decision trees never breastfed group, the only attribute that appeared was receiving an explanation of breastfeeding after delivery.

**Additional file 6. **Attributes selected through machine learning methods. The selected or ranked attributes of the pairs that ever breastfed or never breastfed, considering the class with or without skin-to-skin contact. Receiving an explanation of how to give breast milk or breast to the baby after birth was one of the attributes of greatest importance with respect to the class in both groups.

## Data Availability

The ENADID databases are public and are hosted on the INEGI website, including the design methodology, instrument validation, data collection and analysis [5].

## Abbreviations

BFHI: Baby-friendly Hospital Initiative
INEGI (Spanish acronym): Mexican National Institute of Statistics and Geography
ENADID (Spanish acronym): National Survey of Demographic Dynamics
SD: Standard deviation
UNICEF: United Nations Children’s Fund
WHO: World Health Organization
