# Supplementary material for: Influence of skin-to-skin contact on breastfeeding: results of the Mexican National Survey of Demographic Dynamics, 2018
Source: Int Breastfeed J. 2022 Jul 7;17:49. doi: 10.1186/s13006-022-00489-2 (PMC9261042; doi:10.1186/s13006-022-00489-2)
Supplement: Supplementary file 1 — Additional file 1. Additional definitions of ENADID 2018 variables analyzed and detailed machine learning methodology. [file 13006_2022_489_MOESM1_ESM.docx]

**Additional file 1**

**Additional definitions of ENADID 2018 variables**

Other variables of the ENADID 2018 that were analyzed included the following: age, “How old are you?”; Considering themselves indigenous, “Do you consider yourself indigenous according to your traditions or customs?” Yes or no. Education level, “What is the final year or grade you achieved in the school?” Grouped into none and up to primary or greater than primary. Floor material in the home, “Of what material is most of the floor in the home composed?” Grouped into dirt flooring or cement, firm flooring, wood, mosaic or other covering. Piped water, “Does the home have piped water inside the home/piped water to the plot of land/no piped water at all?” Yes or no. Refrigerator, “Does the home have a refrigerator?” Yes or no. Delivery in a medical unit, “Did the delivery take place in a medical unit?” Yes or no. Delivery type, “Was the delivery normal (vaginal)/programmed cesarean section/emergency cesarean section?” Grouped into vaginal birth or cesarean section.

**Detailed machine learning methodology**

The class (groups) with or without direct skin-to-skin contact immediately after birth and the attributes (characteristics) analyzed were: maternal age, <18 and >40 years or ≥18 years and<40 years. Sociodemographic stratum, low/medium-low or medium-high/high. Locality, rural or urban. Indigenous self-adscription, yes or no. Education level, ≤primary or >primary. Type of delivery, cesarean section or vaginal birth. Receiving an explanation of breastfeeding after delivery, yes or no. Initiation of breastfeeding during the first hour of life, yes or no. Duration of breastfeeding, <6 months or ≥6 months. Motives for not breastfeed/never had milk/ infant rejection or doctor recommended/milk formula/maternal illness/child illness.
