# Supplementary material for: Influence of skin-to-skin contact on breastfeeding: results of the Mexican National Survey of Demographic Dynamics, 2018
Source: Int Breastfeed J. 2022 Jul 7;17:49. doi: 10.1186/s13006-022-00489-2 (PMC9261042; doi:10.1186/s13006-022-00489-2)
Supplement: Supplementary file 2 — Additional file 2. Ever breastfed group Bayesian network, data from the Mexican National Survey of Demographic Dynamics 2018. The analysis through Bayesian networks in mother-baby pairs that ever breastfed, showed a relationship of probabilistic dependence between the skin-to-skin contact and receiving an explanation of breastfeeding after delivery, initiation of breastfeeding and breastfeeding. There is a probabilistic relationship of skin-to-skin contact with delivery type, maternal age, sociodemographic stratum, considering themselves indigenous, locality, and education level. Two attributes converge probabilistically for the initiation of breastfeeding: delivery type and skin-to-skin contact, while four attributes converge for the duration of breastfeeding: sociodemographic stratum, indigenous self-adscription, delivery type and skin-to-skin contact. [file 13006_2022_489_MOESM2_ESM.docx]

Additional file 2. Ever breastfed group Bayesian network, data from the Mexican National Survey of Demographic Dynamics 2018 N=18564


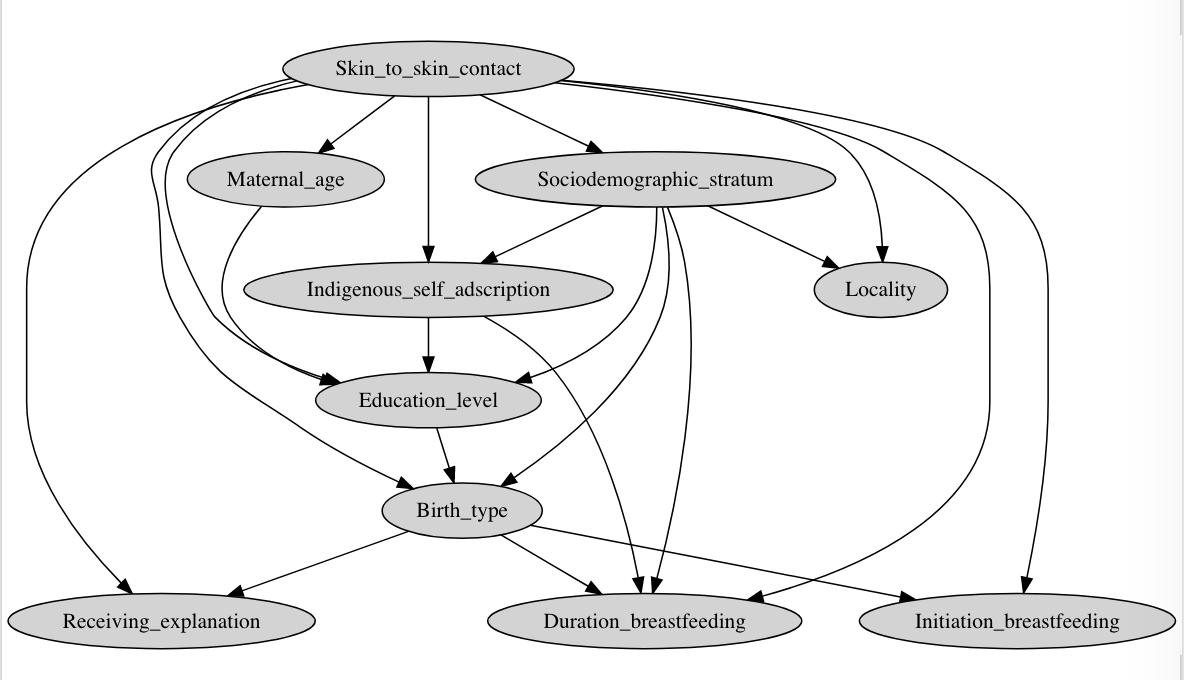


Correctly classified instances 15170 (accuracy 81.7%; sensitivity 97.5%; specificity 12.1%; ROC area 0.693; PRC area 0.795).
