# Supplementary material for: Influence of skin-to-skin contact on breastfeeding: results of the Mexican National Survey of Demographic Dynamics, 2018
Source: Int Breastfeed J. 2022 Jul 7;17:49. doi: 10.1186/s13006-022-00489-2 (PMC9261042; doi:10.1186/s13006-022-00489-2)
Supplement: Supplementary file 3 — Additional file 3. Ever breastfed group decision tree, data from the Mexican National Survey of Demographic Dynamics 2018. Analysis of the decision trees of mother-baby pairs that ever breastfed, had a depth of four levels from the root node, skin-to-skin contact, and 11 nodes, including six terminal nodes. The variable that produces the first split in the decision tree is receiving an explanation of breastfeeding after delivery; initiation of breastfeeding appears at the second division, followed by delivery type and the duration of breastfeeding. [file 13006_2022_489_MOESM3_ESM.docx]

Additional file 3 Ever breastfed group decision tree, data from the Mexican National Survey of Demographic Dynamics 2018 N=18564


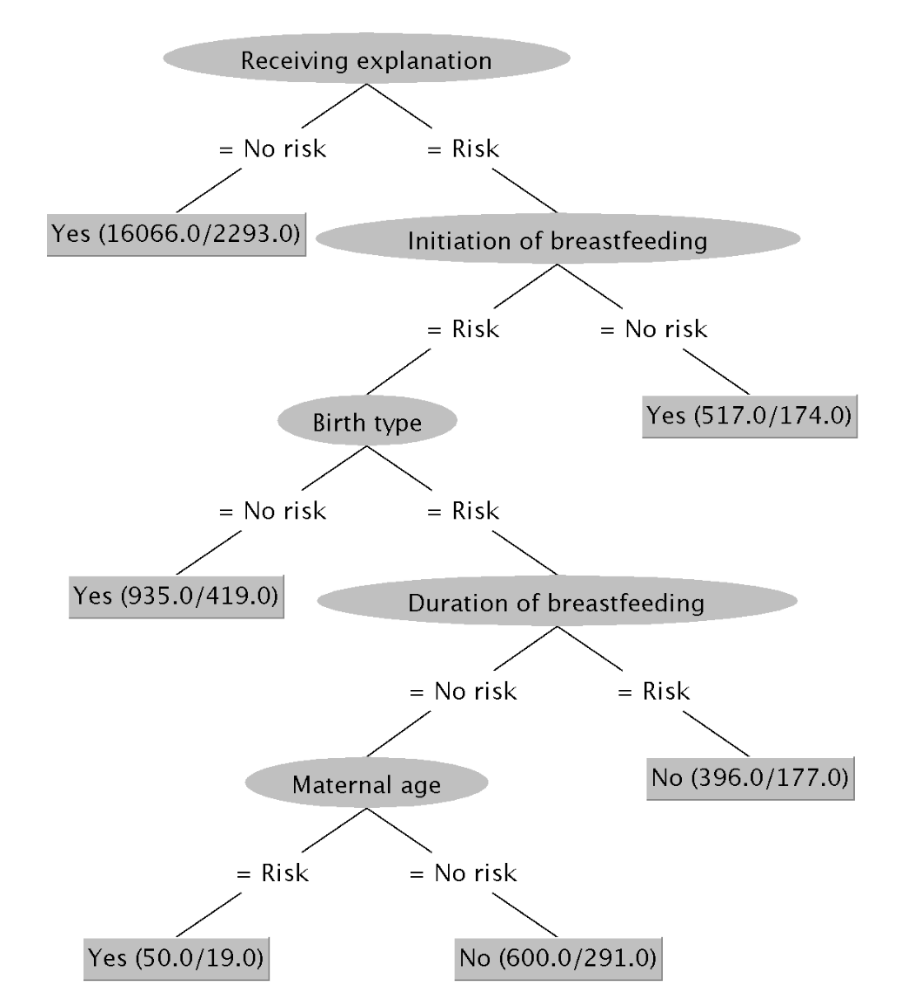


Correctly classified instances 15184 (accuracy 81.8%; sensitivity 97.1%; specificity 14.2%; ROC area 0.618; PRC area 0.753).
