# Supplementary material for: Influence of skin-to-skin contact on breastfeeding: results of the Mexican National Survey of Demographic Dynamics, 2018
Source: Int Breastfeed J. 2022 Jul 7;17:49. doi: 10.1186/s13006-022-00489-2 (PMC9261042; doi:10.1186/s13006-022-00489-2)
Supplement: Supplementary file 4 — Additional file 4. Never breastfed group Bayesian network, data from the Mexican National Survey of Demographic Dynamics 2018. The analysis through Bayesian networks in mother-baby pairs that never breastfed, skin-to-skin contact and receiving an explanation of breastfeeding after delivery were directly related to the motive for no breastfed. [file 13006_2022_489_MOESM4_ESM.docx]

Additional file 4 Never breastfed group Bayesian network, data from the Mexican National Survey of Demographic Dynamics 2018 N=1661.


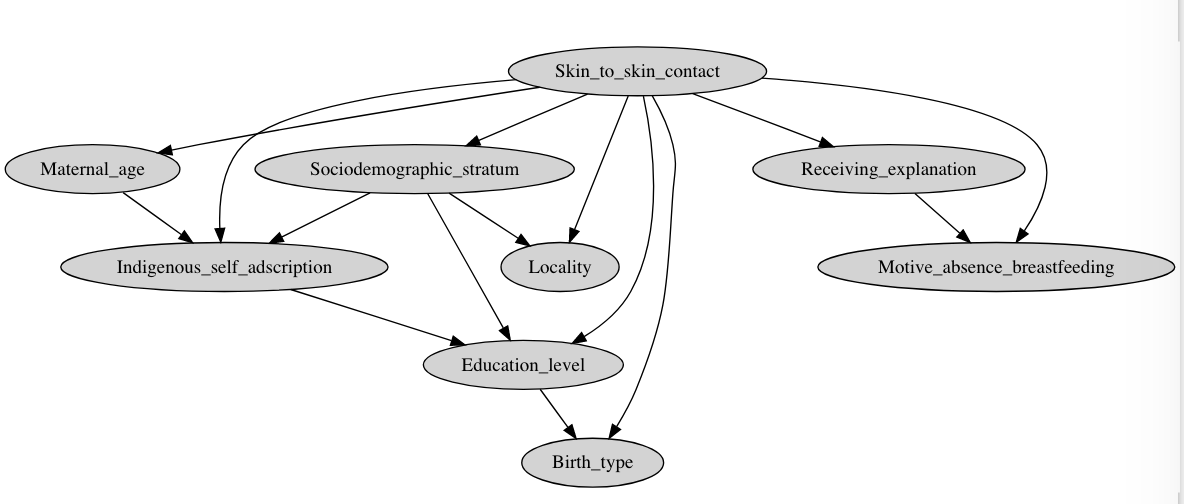


Correctly classified instances 1230 (accuracy 74.1%; sensitivity 92.3%; specificity 35.6%; ROC area 0.703; PRC area 0.720).
