# Supplementary material for: Influence of skin-to-skin contact on breastfeeding: results of the Mexican National Survey of Demographic Dynamics, 2018
Source: Int Breastfeed J. 2022 Jul 7;17:49. doi: 10.1186/s13006-022-00489-2 (PMC9261042; doi:10.1186/s13006-022-00489-2)
Supplement: Supplementary file 5 — Additional file 5. Never breastfed group decision tree, data from the Mexican National Survey of Demographic Dynamics 2018. The analysis of the decision trees never breastfed group, the only attribute that appeared was receiving an explanation of breastfeeding after delivery. [file 13006_2022_489_MOESM5_ESM.docx]

Additional file 5 Never breastfed group decision tree, data from the Mexican National Survey of Demographic Dynamics 2018 N=1661.


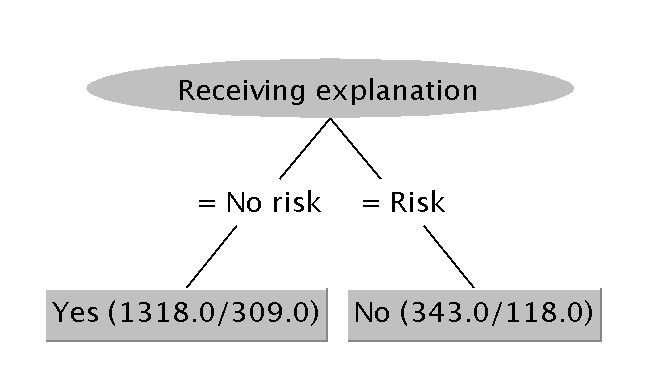


Correctly classified instances 1230 (accuracy 74.1%; sensitivity 89.5%; specificity 41.4%; ROC area 0.643; PRC area 0.666).
