# Supplementary material for: Influence of skin-to-skin contact on breastfeeding: results of the Mexican National Survey of Demographic Dynamics, 2018
Source: Int Breastfeed J. 2022 Jul 7;17:49. doi: 10.1186/s13006-022-00489-2 (PMC9261042; doi:10.1186/s13006-022-00489-2)
Supplement: Supplementary file 6 — Additional file 6. Attributes selected through machine learning methods. The selected or ranked attributes of the pairs that ever breastfed or never breastfed, considering the class with or without skin-to-skin contact. Receiving an explanation of how to give breast milk or breast to the baby after birth was one of the attributes of greatest importance with respect to the class in both groups. [file 13006_2022_489_MOESM6_ESM.docx]

Additional file 6. Evaluation of attributes for the class: with or without skin-to-skin contact, data from the Mexican National Survey of Demographic Dynamics 2018

| **Evaluator of attributes** | **Selected or ranked attributes** | |
| --- | --- | --- |
|  | **Ever breastfed**  **N=18564** | **Never breastfed**  **N=1661** |
| Correlation-based feature subset selection | Delivery type  Receive explanation of breastfeeding | Delivery type  Receive explanation of breastfeeding  Motive for never breastfed |
| Classifier  attribute evaluator | Duration of breastfeeding  Indigenous self-adscription  Sociodemographic stratum | Motive for never breastfed  Receive explanation of breastfeeding  Sociodemographic stratum |
| Correlation attribute evaluator | Receive explanation of breastfeeding Delivery type  Initiation of breastfeeding | Receive explanation of breastfeeding Motive for never breastfed  Delivery type |
| Gain ratio attribute evaluator | Receive explanation of breastfeeding Delivery type  Initiation of breastfeeding | Receive explanation of breastfeeding Motive for never breastfed  Delivery type |
| Information gain attribute evaluator | Receive explanation of breastfeeding Delivery type  Initiation of breastfeeding | Receive explanation of breastfeeding  Motive for never breastfed  Delivery type |
| One-rule attribute evaluator | Duration of breastfeeding  Indigenous self-adscription  Sociodemographic stratum | Receive explanation of breastfeeding  Motive for never breastfed  Sociodemographic stratum |
| Relief-F attribute evaluator | Education level  Initiation of breastfeeding  Maternal age | Receive explanation of breastfeeding  Maternal age  Motive for never breastfed |
| Symmetrical uncertainty attribute evaluator | Receive explanation of breastfeeding  Delivery type  Initiation of breastfeeding | Receive explanation of breastfeeding  Motive for never breastfed |
| Receiving an explanation of breastfeeding after delivery, is listed as the attribute “Receive explanation of breastfeeding”. Only the three most important attributes are shown. | | |
